# Metabotropic Acetylcholine and Glutamate Receptors Mediate PI(4,5)P_2_ Depletion and Oscillations in Hippocampal CA1 Pyramidal Neurons *in situ*

**DOI:** 10.1038/s41598-018-31322-8

**Published:** 2018-08-28

**Authors:** Sandra Hackelberg, Dominik Oliver

**Affiliations:** 1 Institute of Physiology and Pathophysiology, Philipps University, 35037 Marburg, Germany; 2 Present Address: The Ken and Ruth Davee Department of Neurology, Feinberg School of Medicine, Northwestern University, Chicago, IL 60611 USA; 3 DFG Research Training Group, Membrane Plasticity in Tissue Development and Remodeling, GRK 2213, Philipps University, Marburg, Germany; 4 Center for Mind, Brain and Behavior (CMBB), Marburg and Giessen, Germany

## Abstract

The sensitivity of many ion channels to phosphatidylinositol-4,5-bisphosphate (PIP_2_) levels in the cell membrane suggests that PIP_2_ fluctuations are important and general signals modulating neuronal excitability. Yet the PIP_2_ dynamics of central neurons in their native environment remained largely unexplored. Here, we examined the behavior of PIP_2_ concentrations in response to activation of Gq-coupled neurotransmitter receptors in rat CA1 hippocampal neurons *in situ* in acute brain slices. Confocal microscopy of the PIP_2_-selective molecular sensors tubby_CT_-GFP and PLCδ1-PH-GFP showed that pharmacological activation of muscarinic acetylcholine (mAChR) or group I metabotropic glutamate (mGluRI) receptors induces transient depletion of PIP_2_ in the soma as well as in the dendritic tree. The observed PIP_2_ dynamics were receptor-specific, with mAChR activation inducing stronger PIP_2_ depletion than mGluRI, whereas agonists of other Gα_q_-coupled receptors expressed in CA1 neurons did not induce measureable PIP_2_ depletion. Furthermore, the data show for the first time neuronal receptor-induced oscillations of membrane PIP_2_ concentrations. Oscillatory behavior indicated that neurons can rapidly restore PIP_2_ levels during persistent activation of Gq and PLC. Electrophysiological responses to receptor activation resembled PIP_2_ dynamics in terms of time course and receptor specificity. Our findings support a physiological function of PIP_2_ in regulating electrical activity.

## Introduction

Phosphatidylinositol-4,5-bisphosphate (PIP_2_) directly controls many cellular functions, including membrane and cytoskeletal dynamics and the activity of membrane proteins^1–4^. These regulatory effects of PIP_2_ are mediated by modulation of activity or of membrane association of PIP_2_-interacting proteins. In particular, many ion channels are highly sensitive to manipulation of PIP_2_ levels^5,6^.

In cell culture models activation of Gα_q_-coupled receptors can deplete the PIP_2_ content of the plasma membrane by activating phospholipase Cβ (PLCβ)^1–3,7^. Such PIP_2_ concentration changes were also observed in primary cultures of purkinje^8^ and hippocampal^9–12^ neurons. A series of thorough analyses of PIP_2_ signaling in response to muscarinic receptor activity in isolated sympathetic ganglion neurons (SGC)^13–18^, established that in these neurons activation of some Gq-coupled receptors leads to transient depletion of PIP_2_, which in turn inhibits Kv7.2/3-mediated M-currents. Hence, PIP_2_ depletion downstream of receptor-mediated pathways may be a ubiquitous principle controlling neuronal activity by modulating ion channels^19,20^. Thus it seems likely that the well-known increase of excitability by modulatory neurotransmitters, e.g. in hippocampal pyramidal neurons^21,22^, is mediated by deactivation of PIP_2_ -dependent channels^23^. There is also some evidence for PIP_2_-dependent regulation of Kir and K2P channels in striatal and thalamic neurons, respectively^24,25^. However, channel modulation may as well be mediated by other cellular signals downstream of Gq activity. Thus Kv7 channels are also inhibited by intracellular Ca^2+^ elevation^26^ and inhibition of Gq-sensitive TASK channels is mediated by DAG^27^. Both, Ca^2+^ and DAG signals may occur without a substantial drop in PIP_2_ downstream of PLCβ^28–30^.

Another issue is the method used to assess PIP_2_ dynamics. The standard approach is live-cell fluorescence microscopy using genetically encoded sensors built upon PIP_2_-binding domains fused to fluorescent proteins^3^. The most popular sensor domain also used by the studies cited above is the pleckstrin homology domain from PLCδ1 (PLCδ1-PH)^31,32^. However, interpretation of the observations is complicated by the IP_3_ affinity of PLCδ1-PH, compromising its suitability as a sensor of PIP_2_ following PLC activation^33,34^. More recently, the PIP_2_-specific tubby_CT_ domain enabled unequivocal measurement of Gα_q_-induced PIP_2_ depletion in cultured sympathetic and hippocampal neurons^15,33^. While these findings derived from isolated neurons are consistent with substantial PIP_2_ concentration changes, they might still not reflect physiological conditions. Studies in cardiac myocytes showed that PIP_2_ content can considerably differ between isolated cells and cells *in situ*^35^. Besides differences in the extent of PIP_2_ depletion, differences in time course may also have relevance for signaling via this pathway. Thus, knowledge of PIP_2_ dynamics in native neurons is required for understanding their physiological significance.

To address these issues, we characterized the PIP_2_ concentration behavior induced by activation of Gα_q_/PLC-coupled transmitter receptors in hippocampal CA1 pyramidal neurons *in situ* in acute brain slices. These neurons receive modulatory cholinergic input from the septohippocampal pathway^36^, which is mediated postsynaptically by Gq-coupled M1/M3 receptors and results in transient changes of excitability^37–39^. We find that mAChR and mGluRI receptors induce robust PIP_2_ depletion in soma and main apical dendrites. Strikingly, both receptors induced PIP_2_ oscillations. Moreover, PIP_2_ depletion was receptor and neuron type-specific. Correlation with changes in electrophysiological activity supports an instructive signaling role of these neuronal PIP_2_ dynamics.

## Results

### Muscarinic receptors mediate PIP_2_ dynamics in CA1 neurons *in situ*

We began our investigation of PIP_2_ dynamics in acute brain slices by examining the response of hippocampal CA1 pyramidal neurons to activation of their muscarinic ACh receptors. In order to measure Gα_q_ induced PIP_2_ dynamics *in situ*, we expressed genetically encoded PIP_2_ sensors by stereotaxic injection of lentiviral expression vectors into the hippocampi of juvenile (P21) rats (see Methods). Two different GFP-fused sensor domains were used, tubby_CT_-GFP^15,40,41^ and PLCd1-PH-GFP^31,32^. Both sensors work as ‘translocation sensors’, i.e. their degree of membrane association is a direct measure for PIP_2_ concentration and its temporal dynamics^3^.

**Figure 1:**
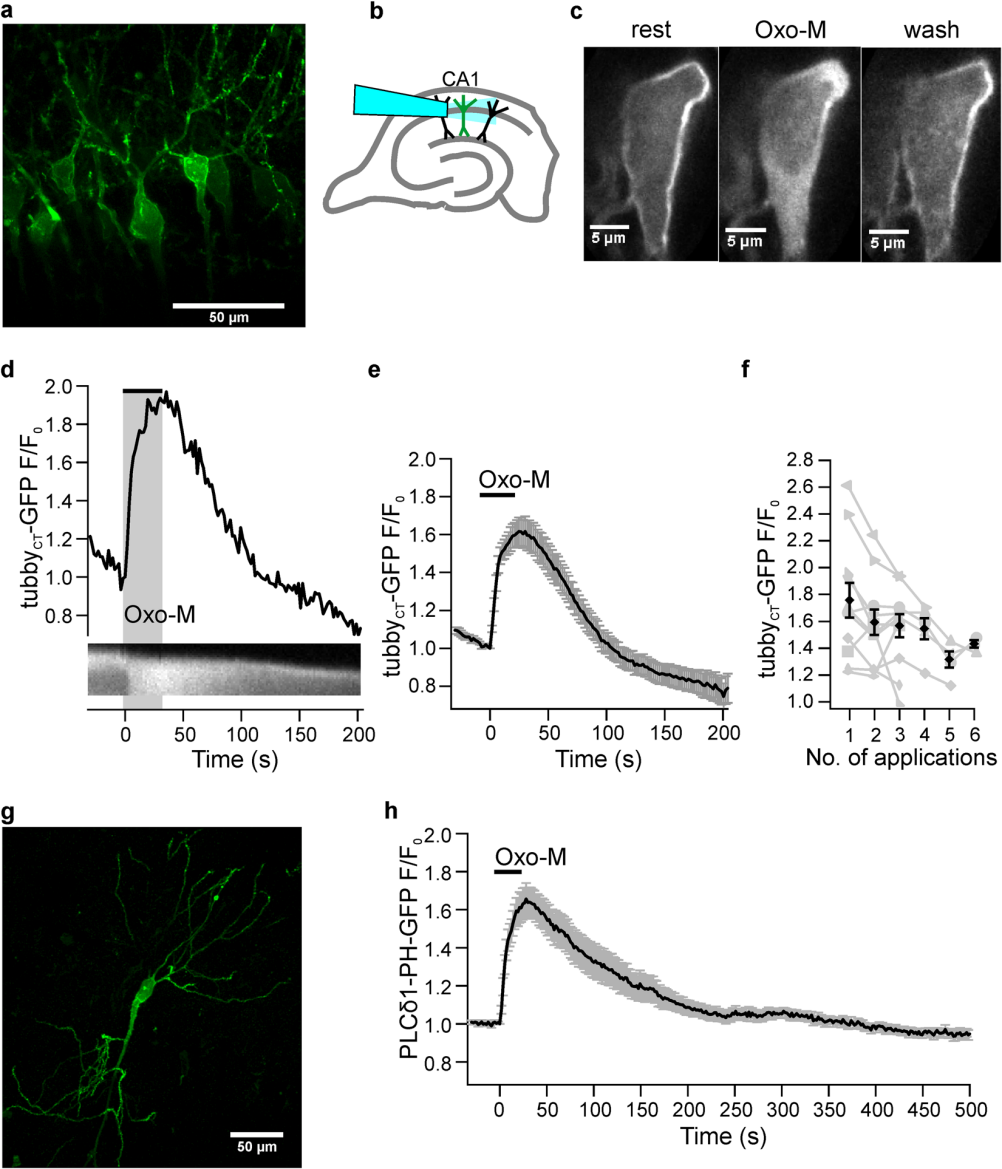
mAChR-mediated PIP_2_ depletion in acute brain slices. (**a**) Representative confocal image of CA1 pyramidal neurons expressing GFP-tagged tubby_CT_ PIP_2_ probes one week after injection of viral vector. (**b**) Schematic diagram of hippocampal slice and positioning of the application capillary in relation to pyramidal neurons in the CA1 area. (**c**) tubby_CT_-GFP translocation during mAChR activation. Under resting conditions probes are associated with PIP_2_ at the membrane. Application of Oxo-M (10 µM) induced probe translocation to the cytosol indicating PIP_2_ depletion, followed by reassociation to the membrane indicating PIP_2_ recovery. (**d**) Representative somatic response to Oxo-M. Shown are cytoplasmatic fluorescence relative to fluorescence before stimulation (F/F_0_, upper panel) and a kymograph of probe translocation between membrane and cytosol (lower panel). (**e**) Average response from 28 individual neurons (26 slices; 16 rats). Application bar is set according to mean response latency. Reduced baseline fluorescence level at the end of the experiments in (**d**,**e**) results from photobleaching of GFP. (**f**) Translocation amplitudes upon repeated Oxo-M application (n = 11, 11, 11, 5, 4, and 3 for successive applications). Responses of individual neurons are shown in grey. (**g**) Confocal image (maximum intensity projection) of a representative CA1 pyramidal neuron expressing PLCδ1-PH-GFP. (**h**) Average translocation of PLCδ1-PH-GFP upon Oxo-M application (n = 16 neurons, 11 slices, 7 rats). Scale bars in (**a**,**f**): 50 µm. Contrast enhancement of 0.4% and 3% was applied to images shown in (**c**,**d**), respectively.

Fluorescence of neurons in acute slices from rats (P26-32) infected with the vector encoding tubby_CT_-GFP indicated successful expression in CA1 pyramidal neurons. As shown in Fig. 1a GFP fluorescence was primarily localized to the plasma membrane of the soma and the dendritic tree.

Activation of mAChR receptors by application of the specific agonist, oxotremorine-M (Oxo-M), induced massive and reversible translocation of the tubby_CT_ probes from the membrane to the cytoplasm in >95% of CA1 pyramidal cell somata examined, indicating strong PIP_2_ depletion (Fig. 1c). To quantify extent and time-course of probe translocation and hence PIP_2_ dynamics we measured fluorescence intensity changes in cytosolic ROIs, as cytosolic signals turned out to be less sensitive to tissue movement than when measuring from the small membrane compartment. Consequently an increase of fluorescence signal corresponds to probe dissociation from the membrane, indicating a loss of PIP_2_. Figure 1d shows a representative response to application of Oxo-M for 30 s. The mean tubby_CT_ translocation reached a peak cytoplasmic amplitude (F/F_0_) of 1.73 ± 0.06 (mean ± SEM; n = 28; 26 slices; 16 rats; Fig. 1e). Mean response latency was 8.8 ± 2.5 s and 90% of the peak response (t_90_) was reached within 19.9 ± 2.4 s. Upon washout of the agonist PIP_2_ levels recovered within about 100 s as indicated by re-association of the probe to the membrane. Cytoplasmic fluorescence returned to 10% of peak amplitude (t_10_, i.e. 90% recovery) in 65.1 ± 7.6 s. To explore the variability of muscarinic PIP_2_ dynamics, Oxo-M was applied repetitively with subsequent stimulations separated by a time interval of >10 minutes (Fig. 1f). On average, the degree of PIP_2_ depletion exhibited a slight but consistent decline in the course of repetitive stimulation. This is consistent with desensitization of muscarinic signaling previously observed in primary hippocampal neurons^11^.

Previous experiments with cultured neurons have used another sensor domain, PLCδ1-PH, to examine PIP_2_ dynamics^9,17,18^. In contrast to the tubby_CT_ sensor, however, PLCδ1-PH has a significant affinity for IP_3_, which is produced whenever PIP_2_ is cleaved by PLCβ^33,34,40,42–44^. Therefore the reliability of PLCδ1-PH as an indicator of PIP_2_ dynamics during GqPCR/PLC signaling has remained an unresolved issue^33,34^, which provided the rationale for choosing tubby_CT_ in the present study. Indeed, previous studies showed considerable differences in the behavior of tubby_CT_-GFP and PLCδ1-PH-GFP sensors in terms of translocation following PLC activation^15,34,40^. Thus, we were interested in comparing both sensors in acute brain slices. When PLCδ1-PH-GFP was expressed in CA1 neurons (Fig. 1g), stimulation of mAChRs resulted in robust translocation of fluorescence in all neurons examined (Fig. 1h), similar to the results obtained with tubby_CT_-GFP (PLCδ1-PH-GFP: latency 4.9 ± 1.2 s; t_90_ 22.3 ± 1.9 s; F/F_0 = _1.72 ± 0.07; n = 16, 11 slices, 7 rats). However, the recovery was significantly slower compared to tubby_CT_-GFP (t_10_ = 132.7 ± 20.8 s; t-test p = 0.008), suggesting that responses of the PH sensor are co-determined by IP_3_ production.

### Receptor-specific PIP_2_ depletion in CA1 neurons

**Figure 2:**
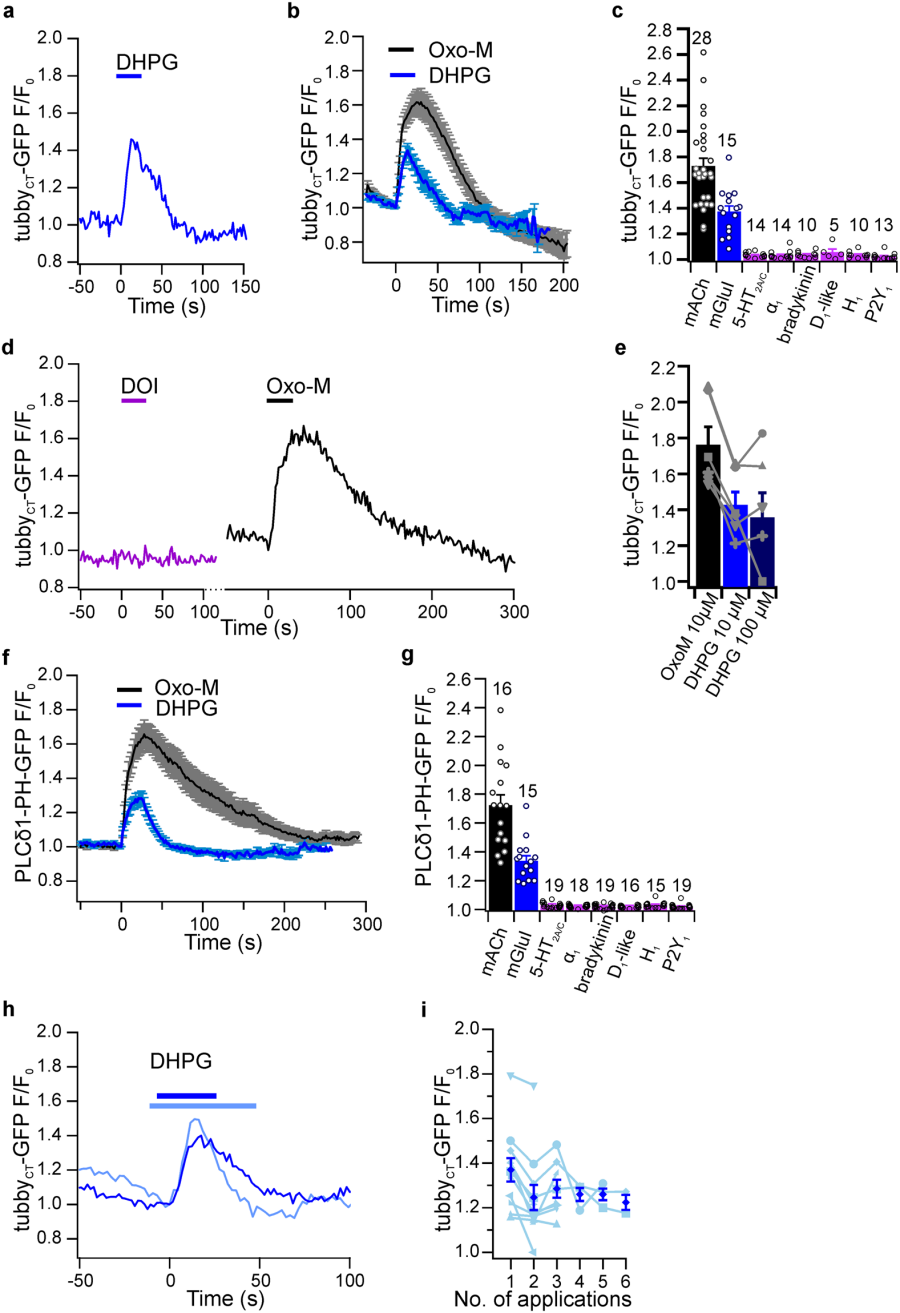
Somatic PIP_2_ dynamics are receptor specific. (**a**) Representative experiment showing PIP_2_ depletion in a CA1 soma in response to application of mGluRI agonist DHPG (10 µM) as determined by tubby_CT_-GFP translocation. (**b**) Average PIP_2_ concentration changes obtained from 15 cells as in (**a**) (blue). Response to activation of mAChRs (grey) is redrawn from Fig. 1e for comparison (n = 28). (**c**) Summary of peak PIP_2_ depletion (translocation of tubby_CT_-GFP) upon application of specific agonists for the Gq-coupled receptors indicated. Agonists applied were Oxo-M (10 µM, n = 28 neurons/26 slices/16 rats), DHPG (10 µM, n = 15/14/11), DOI, 10–20 µM, n = 14/14/6), methoxamine (10–20 µM; n = 14/14/6), bradykinin (10–20 µM, n = 10/10/4), SKF 83959 (10–20 µM, n = 5/5/3), 2-pyridylethylamin (10–50 µM, n = 10/10/4), ADPβS (10 µM; application for 30 to 60 s, n = 13/13/4). Numbers of experiments also indicated above bars. (**d**) Representative sensor responses measured from the same CA1 neuron during successive application of 5-HT_2A/2C_ agonist DOI and mAChR agonist Oxo-M. (**e**) Response to higher concentration (100 µM) of DHPG did not increase tubby_CT_-GFP translocation (application for 5 min each; n = 6, 6 slices, 3 rats). Successive responses of individual neurons shown in grey. (**f**) Average response of PLCδ1-PH-GFP sensor to mGluRI activation (blue, DHPG) and mAChR activation (black; Oxo-M; replotted from Fig. 1h). (**g**) Peak translocation of PLCδ1-PH-GFP sensor during application of agonists as in (**c**); Oxo-M (n = 16 neurons, 11 slices, 7 rats), DHPG (n = 15/11/7), DOI (n = 19/13/7), methoxamine (n = 18/12/7), bradykinin (n = 19/13/7), SKF 83959 (n = 16/11/6), 2-pyridylethylamin (n = 15/11/6), ADPβS (n = 19/13/7). (**h**) Example responses from two different neurons to application of DHPG (10 µM). Note that one response (dark blue) closely matches the average response while the other (light blue) shows pronounced recovery in the presence of the agonist. (**i**) Responses to repeated DHPG application (n = 11, 11, 8, 3, 3, and 2 for successive applications, respectively; delay between applications ≥ 10 min; individual responses indicated in light blue).

Activation of PLCβ and subsequent hydrolysis of PIP_2_ to IP_3_ and DAG is the main signaling pathway of Gα_q_-coupled receptors. Yet it is not known if PIP_2_ depletion is generally associated with the activation of Gα_q_ coupled receptors other than M1/M3 receptors in central neurons. For example, in sympathetic ganglion neurons activity of muscarinic and purinergic receptors results in a depletion of PIP_2_, whereas bradykinin receptors generate IP_3_-dependent Ca^2+^ signals without substantial changes of the PIP_2_ concentration^17,18,29,45,46^. CA1 pyramidal neurons express various Gα_q_-coupled receptors which could potentially induce PIP_2_ depletion, including group I metabotropic glutamate receptor^47^, α_1A_-adrenoreceptor^48^, bradykinin B_2_ receptor^49^, Gq-coupled dopamine_D1-like_^50–55^, histamine H_1_ receptor^56,57^, P2Y_1_ receptor^58,59^, and 5-HT_2A/2C_ receptors^60–62^. We applied specific agonists of group I mGluRs, 5-HT_2A/2C_ receptors α_1A_ adrenoreceptors, bradykinin receptors, Gq-coupled dopamine_D1-like_ receptors, H_1_ histamine receptor, or P2Y_1_ receptor and monitored PIP_2_ concentrations with tubby_CT_-GFP. Of these receptors, only mGluRs induced detectable probe translocation indicative for depletion of PIP_2_ (Fig. 2a). mGluRI-induced PIP_2_ depletion in neuronal somata was consistent across the population of neurons examined (F/F_0_ = 1.37 ± 0.05; n = 15; 14 slices from 11 rats; Fig. 2b).

As summarized in Fig. 2c, PIP_2_ levels in CA1 neurons were insensitive to activation of any of the other receptors. Importantly, subsequent control application of Oxo-M triggered robust translocation of tubby_CT_-GFP in each neuron, indicating proper responsiveness of the cell and appropriate sensitivity of the detection approach (Fig. 2d). Further, the prolonged application of each of the agonists (except muscarinic and glutamatergic) for up to 120 seconds or application of the endogenous ligands serotonin and dopamine did not evoke detectable responses (not shown). Equivalent results were obtained with neurons expressing the alternative PIP_2_ sensor domain, PLCδ1-PH-GFP. As shown in Fig. 2f and g, the stimulation of mAChR and mGluRI but none of the other receptors examined induced translocation of PLCδ1-PH-GFP. In summary, results obtained with both sensor domains indicate that α_1A_-adrenoreceptor, bradykinin, dopamin_D1-like_, histamin-H_1_, P2Y_1_, and 5-HT_2A/2C_ do not induce significant PIP_2_ depletion in the soma of CA1 pyramidal neurons. Thus, PIP_2_ depletion is specific to mAChR and mGluRI, at least in the context of standard experimental conditions. However, response magnitude of mGluR activation was significantly lower compared to muscarinic PIP_2_ depletion in a population of cells challenged by both agonists (n = 12, 11 slices, 8 rats; paired t-test p = 0.032). While Oxo-M and DHPG have similar binding affinities for their cognate receptors^63^, EC50 values for downstream effects such as Ca^2+^ responses are often higher for DHPG than for Oxo-M, raising the possibility that 10 µM of DHPG might not be sufficient to evoke a saturating PIP_2_ response. However, increasing the concentration to 100 µM or the duration of agonist application of the glutamatergic agonist (DHPG) did not further increase the response mediated by mGluRs (Fig. 2e) and these responses were significantly smaller than muscarinic responses in the same neurons (p < 0.05, one way ANOVA followed by Tukey post-hoc test, n = 6, 6 slices, 3 rats).

In addition to the smaller responses, mGluRI-induced depletion of PIP_2_ also differed in its time course compared to muscarinic stimulation (Fig. 2a,b,f). As measured with tubby_CT_-GFP, response latencies (6.81 ± 0.89 s) and rise time (t_90_ = 12.68 ± 1.15 s) where comparable, but time course of recovery was faster compared to mAChR activation (t_10_ = 28.38 ± 3.57 s; t-test p = 0.0001) Remarkably, in 6 out of 15 recordings, PIP_2_ levels recovered in the continued presence of the agonist DHPG, as illustrated by individual recordings shown in Fig. 2h. Similarly, PIP_2_ dynamics as measured with the PLCd1-PH-GFP probe showed a much faster recovery after activation of mGluRI (t_10_ = 30.44 ± 2.05 s, n = 15, 14 slices, 7 rats) when compared to mAChRs (t_10_ = 132.74 ± 20.77 s; t-test p = 0.0003; Fig. 2f). As noted with mAChR activation, PIP_2_ dynamics induced by mGluRIs showed slight desensitization in response to repeated application of the agonist (Fig. 2i).

### Dendritic PIP_2_ dynamics

Next, we were interested in the spatial pattern of PIP_2_ depletion, in particular with respect to dendritic compartments. Because we probed PIP_2_ dynamics with translocation sensors that require microscopic resolution of membrane versus cytoplasm the measurements were confined to dendrites with a diameter of more than 1 µm that were localized close to the slice surface allowing for good optical access. Thus we achieved recordings from the main apical dendrite and its major branches up to 300 µm and basal dendrites to 20 µm distal to the soma. The distance between *stratum pyramidale* to *fissura hippocampi* defining the total length of apical dendrites was about 400 µm for our slices.

**Figure 3:**
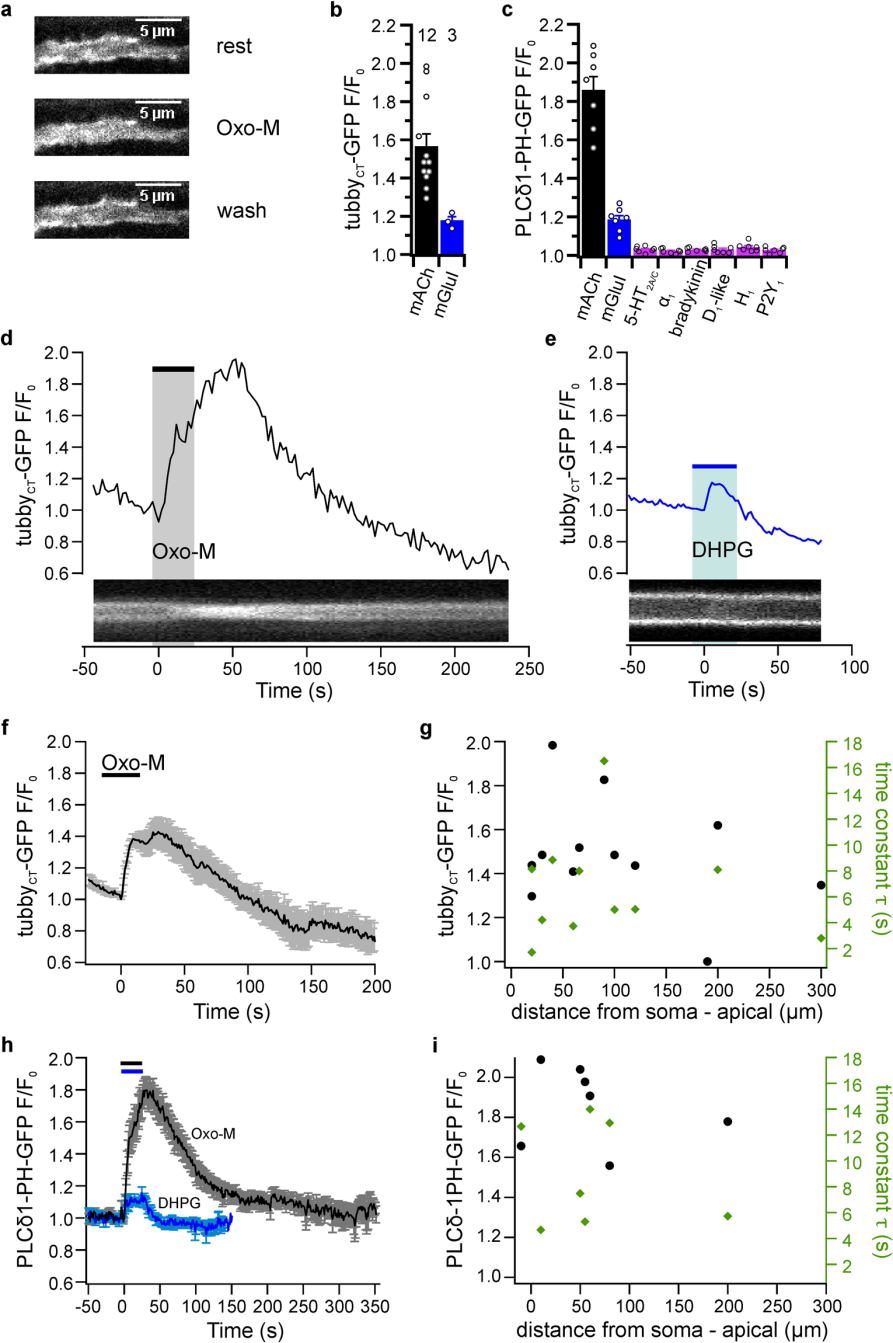
Dendritic PIP_2_ dynamics. (**a**) Translocation of tubby_CT_-GFP in response to Oxo-M application (10 µM) in an apical dendrite 60 µm from the soma. (**b**) Translocation of tubby_CT_-GFP from dendritic membranes induced by mAChR (Oxo-M, 10 µM, n = 12 neurons/12 slices/10 rats) and mGluRI (DHPG, 10 µM, n = 3/3/2) activation, quantified as changes of axial (cytoplasmic) fluorescence changes relative to pre-stimulus fluorescence. (**c**) Translocation of PLCδ1-PH-GFP from dendritic membranes in response to activation of various Gq-coupled receptors. Agonists used as in Fig. 2 (n = 7 neurons, 7 slices, 3 rats for each condition). (**d**) Time course of PIP_2_ depletion from a dendritic recording 220 µm from the soma, shown as the relative increase of axial fluorescence (upper panel) and corresponding kymograph (lower panel). (**e**) Representative fluorescence change and kymograph of dendritic tubby_CT_-GFP translocation in response to application of DHPG (10 µM; apical dendrite; 20 µm from soma). (**f**) Average time course of mAChR-induced dendritic PIP_2_ dynamics. (**g**) Amplitudes (black) and time constants (green) of tubby_CT_-GFP translocation in response to application of Oxo-M plotted as a function of the distance of the recording location from the neuronal soma. Data from 13 individual neurons. (**h**) Average time course of dendritic PLCδ1-PH-GFP sensor translocation in response to application of the agonists indicated (n = 7 each). (**i**) Properties of translocation of PLCδ1-PH-GFP in response to Oxo-M plotted as a function of dendritic recording site relative to soma. Contrast enhancement of 10%, 1% and 3% was applied to images shown in a, d and e, respectively.

We found robust but receptor-specific PIP_2_ depletion in all dendritic compartments examined. Figure 3a shows a representative example illustrating membrane localization of tubby_CT_-GFP in a dendrite and its transient redistribution into the dendritic cytosol during pharmacological activation of mAChRs, indicating reversible depletion of PIP_2_. With the exception of one apical dendrite (190 µm distance from soma), all dendrites examined (n = 12) responded with the translocation of tubby_CT_-GFP. The time course of representative mAChR-induced dendritic PIP_2_ dynamics is further shown in Fig. 3d as a kymograph and quantitatively as the change of cytosolic fluorescence intensity. The average peak amplitude derived from dendrites of 12 neurons was F/F_0_ = 1.57 ± 0.06 (12 slices, 10 rats). The average latency of the responses was 14.29 ± 5.26 s. We observed a rise time t_90_ of 17.52 ± 2.67 s and 90% recovery time (t_10_) was 41.27 ± 7.25 s after the end of the application. As shown in Fig. 3a,e, activation of mGluRI also resulted in translocation of tubby_CT_-GFP. However, the degree of PIP_2_ depletion was significantly weaker compared to activation of mAChRs (F/F_0_ = 1.18 ± 0.02; paired t-test, p = 0.041, n = 3 dendrites in 3 slices from 2 rats. Similar observations were made with PLCδ1-PH-GFP as the PIP_2_ probe (Fig. 3c,h). Thus, activation of muscarinic receptors by Oxo-M induced strong translocation (F/F_0_ = 1.86 ± 0.07; t_90_ = 21.86 ± 1.90 s; t_10_ = 142 ± 30.53 s; n = 7 dendrites, 7 slices, 3 rats), whereas activation of mGluRI receptors by DHPG induced small yet reproducible translocation of PLCdδ1-PH-GFP (F/F_0_ = 1.19 ± 0.02; n = 7). Stimulation of various other Gq-coupled receptors did not induce detectable translocation of PLCδ1-PH-GFP (Fig. 3c).

We wondered about spatial, basal-to-apical signaling gradients along the dendrites. Figure 3g,i display amplitudes and time constants of PIP_2_ depletion as a function of the distance from the soma. We find that neither parameter shows an evident trend along the dendritic distance, suggesting that PIP_2_ depletion is mostly homogenous throughout the larger dendritic compartments examined here (Pearson correlation coefficients: tubby_CT_-GFP amplitudes, −0.071; tubby_CT_-GFP time constants, 0.168;, PLCδ1-PH-GFP amplitudes, −0.209;, PLCδ1-PH-GFP time constants, −0.233).

Taken together, these results indicate that receptor-induced PIP_2_ dynamics in the larger dendritic compartments amenable to examination with translocation sensors is similar to the somatic dynamics. Specifically, activation of muscarinic receptors resulted in strong depletion of PIP_2_, glutamatergic receptors were considerably less effective, and PIP_2_ levels recovered rapidly.

### Prolonged receptor activation revealed complexity of PIP_2_ dynamics

**Figure 4:**
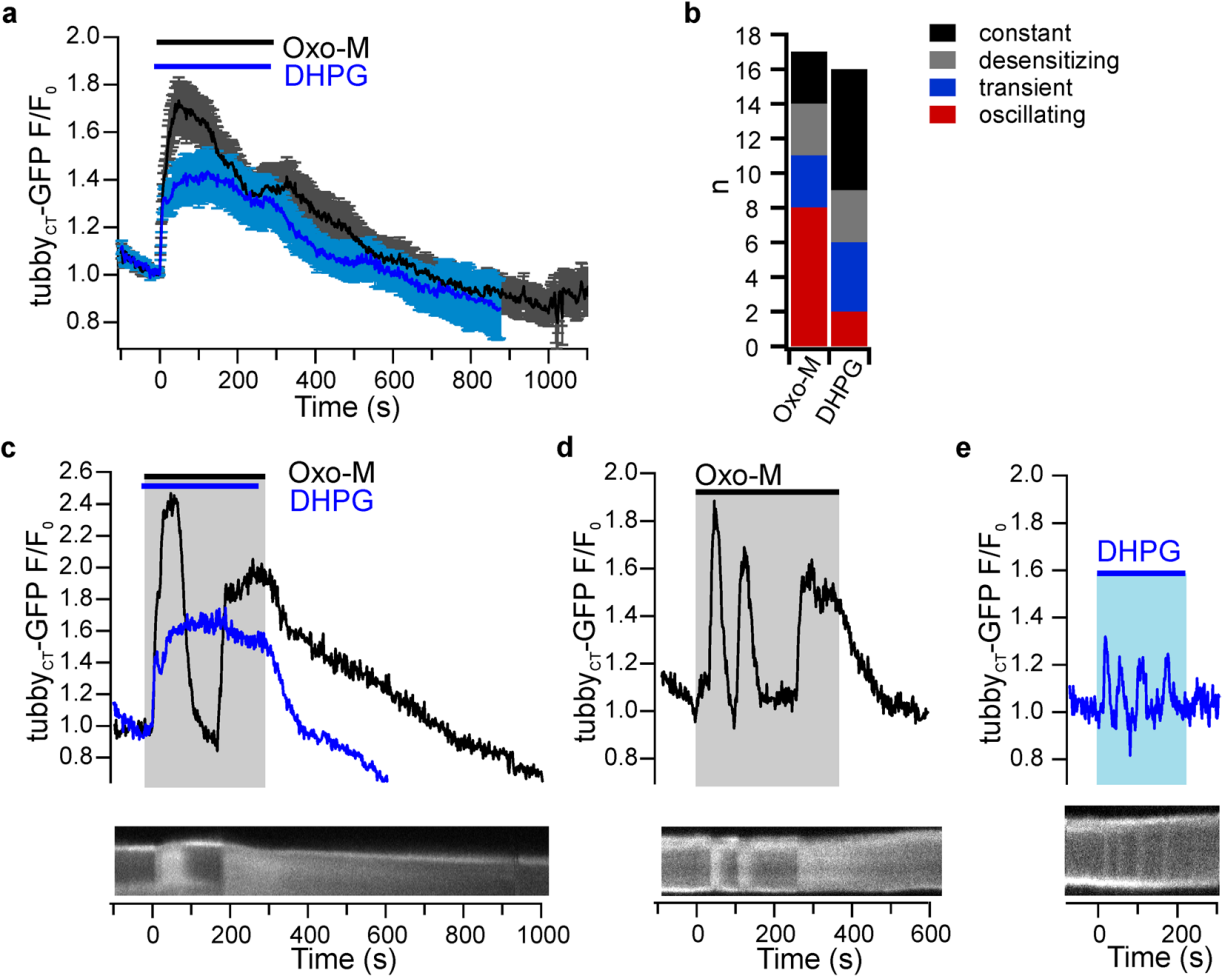
Prolonged receptor activation reveals PIP_2_ oscillations. (**a**) Average time course of somatic PIP_2_ dynamics during extended application of mAChR and mGluRI agonists Oxo-M (n = 17 neurons/15 slices/5 rats) and DHPG (n = 16/14/5) as measured by translocation of tubby_CT_-GFP. (**b**) Distribution of distinct temporal patterns of tubby_CT_-GFP sensor translocation within the population of neurons challenged by sustained application (5 min) of agonists. Responses were categorized as constant, desensitizing (partial recovery), transient (full recovery) and oscillating (≥2 peaks). (**c**) Distinct temporal behavior of PIP_2_ dynamics of an individual pyramidal neuron to successive applications of Oxo-M and DHPG. Lower panel shows a kymograph illustrating the response to Oxo-M. (**d**) Oscillatory PIP_2_ dynamics in a CA1 neuron in response to prolonged activation of mACh receptors. Note the pronounced repetitive phases of rapid PIP_2_ depletion and replenishment during stimulation. (**e**) Oscillatory PIP_2_ dynamics of a neuron in response to continuous activation of mGluRs. Contrast enhancement of 0.4%, 1% and 5% was applied to kymographic images shown in c, d and e, respectively.

The occasionally observed early recovery of PIP_2_ level during activation of mGluRI prompted us to examine the time course of PIP_2_ dynamics during sustained stimulation. Figure 4 shows the resulting PIP_2_ dynamics measured with the tubby_CT_ sensor in neuronal somata during continuous application of the receptor agonists for 5 min. Notably, PIP_2_ depletion generally showed a phasic-tonic time course with an initial strong PIP_2_ depletion followed by partial recovery in the sustained presence of the agonist as apparent from the average from a larger number of neurons (n = 17 and 16 for mAChR and mGluRI activation, respectively; Fig. 4a). The initial rate of PIP_2_ decrease was similar for both receptors (t_90_ = 39.3 ± 5.3 s and 43.3 ± 8.8 s, respectively), but as previously seen with brief receptor stimulation (Fig. 2), the muscarinic responses had a higher average amplitude compared to glutamatergic responses (F/F_0_ = 1.84 ± 0.08 and 1.55 ± 0.07, respectively; paired t-test p = 0.0002, n = 16, 14 slices; 5 rats). PIP_2_ recovery after muscarinic receptor activation was more pronounced than for mGluR stimulation such that PIP_2_ levels tended towards similar values at the end of receptor stimulation period. For both receptors, recovery of PIP_2_ levels after removal of the receptor agonist was slower than observed with brief receptor stimulation (mAChR: t_10_ 243.2 ± 43.9 s; mGluRI: t_10_ 167.8 ± 41.5 s; cf. Fig. 2b) and thus depended on the duration of receptor activation.

While demonstrating partial desensitization of PIP_2_ responses as a common pattern, the prolonged stimulation also revealed considerable variability and complexity in the time course of PIP_2_ dynamics (Fig. 4b). Most strikingly, PIP_2_ depletion was often multiphasic or oscillatory. Examples for such complex PIP_2_ dynamics are shown in Fig. 4c–e. In these cells, PIP_2_ levels returned to baseline despite sustained presence of the agonist, and moreover, multiple depletion events occurred in rapid succession (Fig. 4d,e). Altogether, 8 out of 17 neurons showed oscillatory PIP_2_ concentration dynamics with Oxo-M and two out of 16 during application of DHPG. These observations suggest that PIP_2_ dynamics may be subject to complex temporal regulation and indicate potent PIP_2_ resynthesis capability of CA1 neurons during receptor activation.

### Modulation of electrical behavior by receptor stimulation

The well-described effects of muscarinic activity on the electrical properties of CA1 neurons – including inhibition of M-currents – may (at least partially) be mediated by PIP_2_ concentration dynamics. We thus were interested in differential effects on excitability of the various Gq/PLC-coupled receptors examined for their coupling to PIP_2_ dynamics.

**Figure 5:**
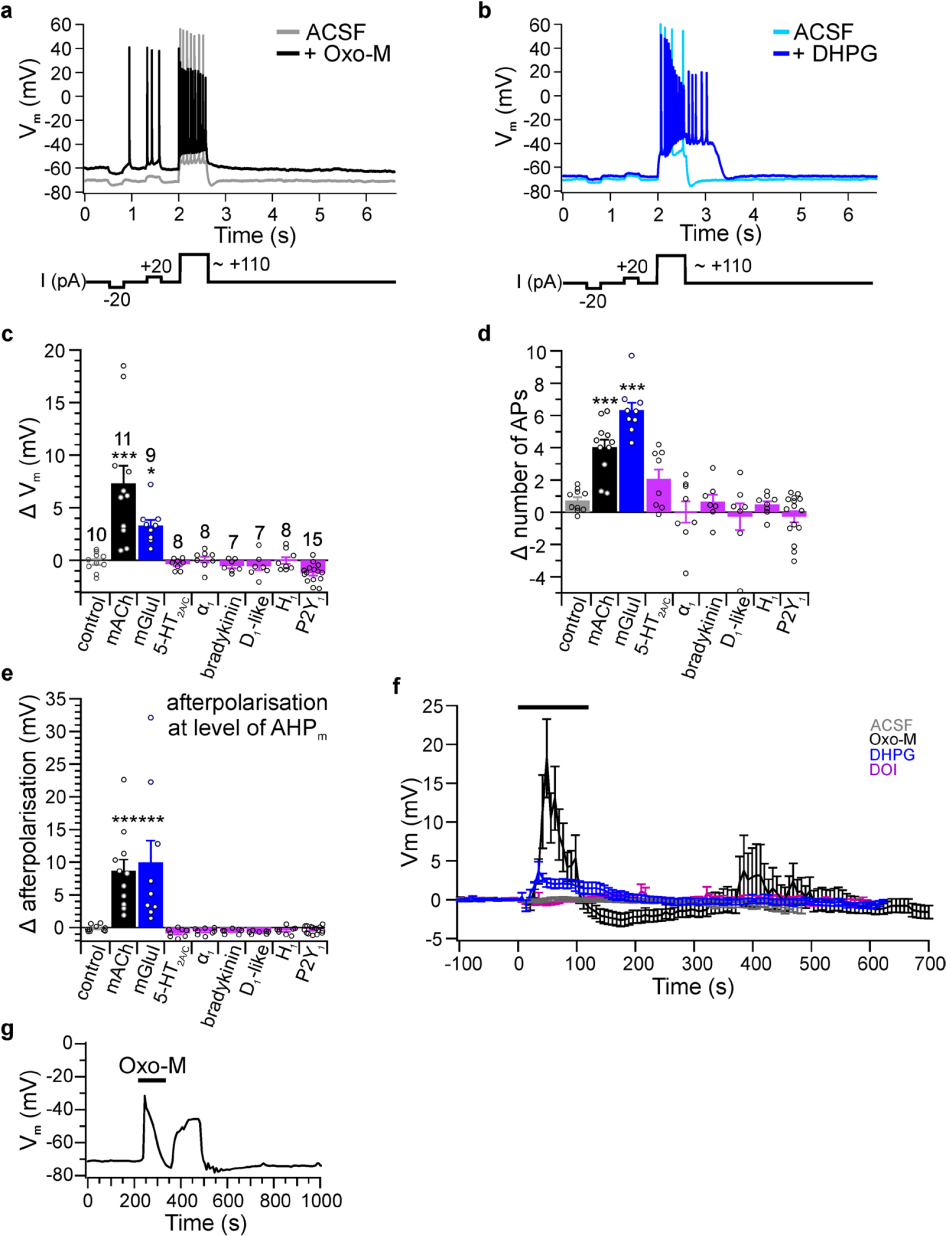
Electrophysiological responses to activation of Gq-coupled receptors. (**a**,**b**) Current clamp protocol (lower panel) and representative recordings of corresponding membrane potential (V_m_) responses of CA1 pyramidal neurons before (light traces) and during (dark traces) activation of mAChRs or mGluRIs by 10 µM Oxo-M or DHPG, respectively. (**c**) Changes of resting membrane potential (ΔV_m_) displayed as the difference before and upon application of various GqPCR activators. Agonists applied were Oxo-M (10 µM; n = 10 neurons/10 slices from 7 rats; data points with plateau potentials were excluded for this analysis) for AChRs, DHPG (10 µM; n = 9/8/5) for mGluRIs, DOI (20 µM; n = 8/8/7) for 5-HT_2A/C_, methoxamine (20 µM; n = 8/8/7) for α_1_-adrenergic receptors, bradykinin (20 µM; n = 7/7/6), SKF 83959 (20 µM; n = 7/7/6) for D1-like dopamine receptors, 2-pyridylethylamin (20 µM; n = 8/8/7) for H_1_ histamine receptors, ADPβS (10 µM; n = 15/15/11) for P2Y_1_-R. Asterisks indicate significance of difference to control application of ACSF (n = 10/10/7) with p < 0.05 (*), 0.01 (**) and 0.001 (***; one-way ANOVA followed by Dunnett multiple comparison test). (**d**) Difference in number of action potentials (AP) triggered during 600 ms depolarizing current step. Numbers of experiments as in (c). (**e**) Changes in afterpolarisation where a negative Δ indicates an AHP_m_ increase and positive Δ indicates an AHP reduction or afterdepolarisation. Numbers of experiments as in (c). (**f**) Mean time courses of V_m_ modulation during application of ACSF as a control (grey), Oxo-M (black), DHPG (blue), and DOI (purple). Vm was measured at the first 500 ms of each trace in the absence of spiking; see (a). (**g**) Example membrane voltage oscillation during mAChR activation in a P27 slice.

To this end, we performed patch clamp experiments in current clamp mode in acute brain slices prepared from rats at P14 to P21. Current step protocols were used to assess membrane potential, input resistance, spiking behavior, and afterpolarisation (Fig. 5a,b). Experiments were performed in the presence of inhibitors of GABA_A/B_ and ionotropic glutamate receptors (see Methods) to exclude effects resulting from network activity.

Overall, we found pronounced changes in electrical behavior following the activation of muscarinic mAChR receptors and mGluRI but little effects of other Gq-coupled receptors. Consistent with previous findings^64–67^ agonists of both mAChR (n = 11, 11 slices, 10 rats) and mGluRI (DHPG 10 µM, n = 9, 8 slices, 5 rats) induced depolarization of the resting membrane potential and an increase in firing frequency during depolarization (number of action potentials, NAP; Fig. 5a–d,f). In CA1 cells, a train of action potentials is usually followed by an afterhyperpolarization (AHP)^37,68,69^. Application of either Oxo-M or DHPG resulted in the disappearance of the AHP and the appearance of an afterdepolarisation (ADP; Fig. 5a,b,e). All of these receptor-induced changes are consistent with the deactivation of potassium conductances such as M currents^37,68,70,71^. In some neurons, activation of mAChR receptors induced sustained depolarization (plateau potentials) subsequent to the 600 ms current step, as also described previously^72^.

As shown in Fig. 5c–f, stimulation of other Gq-coupled receptors, including 5-HT_2A/C_ receptors, α_1_ adrenergic receptors, bradykinin receptors, D_1_-like dopamine receptors, H_1_ histamine receptors, and purinergic P2Y_1_ receptors had no significant effects on either of these electrophysiological characteristics. Noteworthy, the mAChR and mGluRI induced depolarization did not always persist during the agonist application, as occasionally initial transient hyperpolarisation and oscillations of the membrane potential was observed during mAChR activation. During muscarinic stimulation, 9 of 11 neurons showed substantial recovery from depolarization in the presence of Oxo-M; of those, 6 neurons showed complete repolarization or even hyperpolarisation before washout. In the presence of DHPG, 3 neurons showed a substantial recovery from initial depolarization. Oscillatory behavior was observed in neurons from younger animals (P14-21), but also in slices age-matched to the PIP_2_ imaging experiments, as shown in an exemplary recording (Fig. 5g) obtained from a neuron in a P27 slice.

In summary, we find that pronounced changes in membrane potential and firing rates paralleled neuronal PIP_2_ depletion in terms of effect size, time course and receptor specificity. Thus, depolarization, increased spike rates and PIP_2_ dynamics were largely restricted to the activation of mAChR and mGluRI receptors.

### Neuron type-specific PIP_2_ dynamics: dentate gyrus granule cells

**Figure 6:**
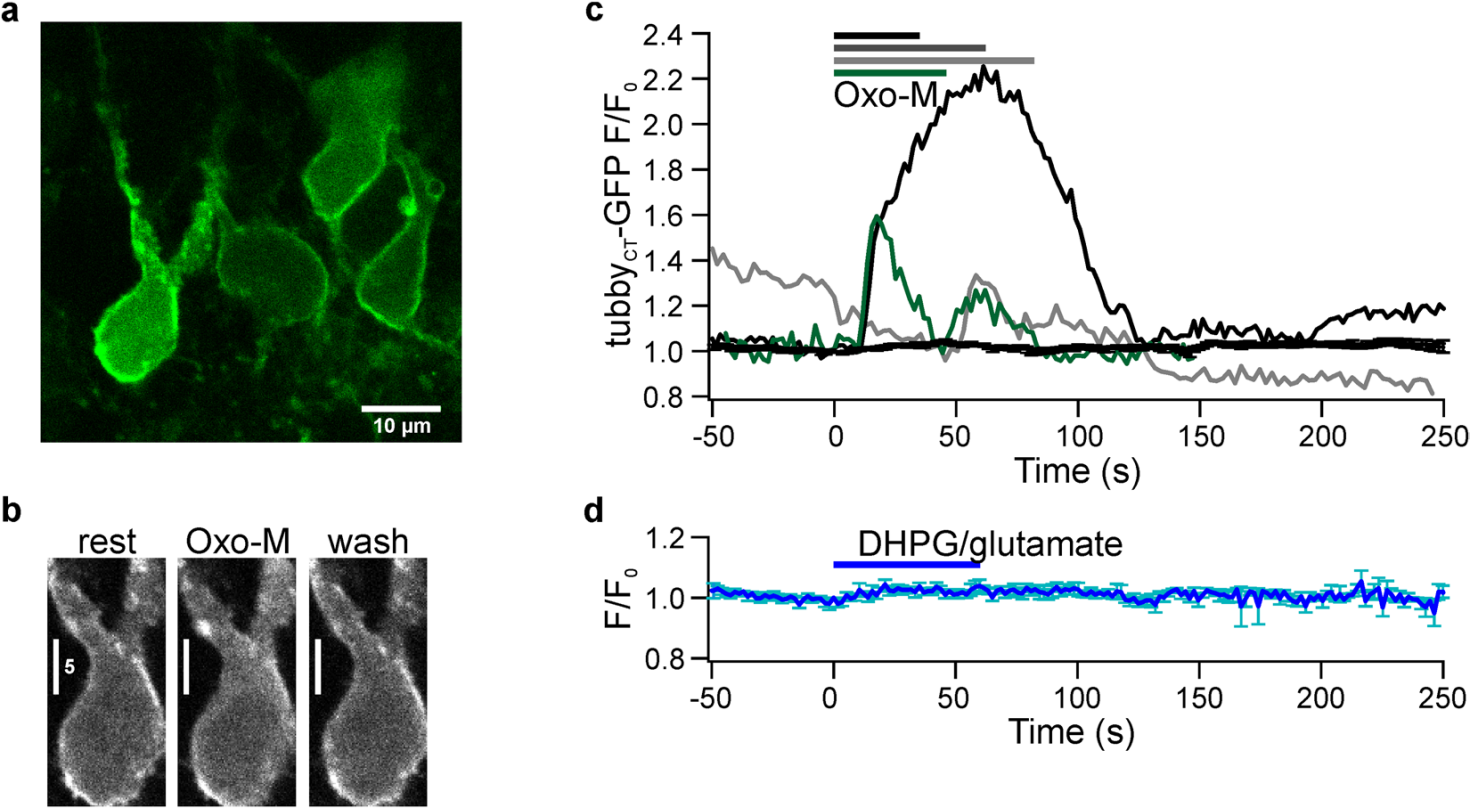
Dentate gyrus granule neurons largely lack receptor-induced PIP_2_ depletion. (**a**) Confocal image of gyrus dentatus granule neurons in an acute hippocampal slice, expressing tubby_CT_-GFP after sterotactic injection of lentiviral vector. (**b**) Confocal images demonstrating a minor degree of probe translocation (1% contrast enhanced; first neuron from left in (**a**)). (**c**) Only 3 out of n = 24 neurons showed mAChR-induced PIP_2_ depletion, as indicated by sensor translocation (3 individual neurons from separate animals). Black trace shows mean fluorescence time course of 21 non-responsive neurons. Time 0 indicates start of the application shown by bars. (**d**) Application of the mGluRI agonist DHPG (n = 2 neurons, both of which were responsive to Oxo-M) or glutamate (n = 4) did not induce PIP_2_ depletion.

To extend our observations on PIP_2_ dynamics to additional neuronal cell types, we measured PIP_2_ dynamics following activation of the same receptors (mAChR and mGluRI) in dentate gyrus granule neurons (Fig. 6a). Granule neurons express both mAChR and mGluRI receptors at the soma^47,73,74^. However, detectable depletion of PIP_2_ upon stimulation with Oxo-M was observed in only three independent experiments (n = 24; Fig. 6b,c). None of the six neurons stimulated with DHPG (10 µM, n = 2) or Glutamate (100 µM, n = 4) showed any detectable sensor translocation (Fig. 6d). Thus, the induction of PIP_2_ dynamics may be highly specific between different types of neurons and this specificity seems to be dependent on mechanisms other than the expression of Gq/PLC-coupled transmitter receptors.

## Discussion

### Direct observation of PIP_2_ dynamics in central neurons *in situ*

While there is good evidence for PIP_2_ depletion in response to activation of Gq-coupled receptors for some types of neurons studied in the cell culture dish, surprisingly little is known about the prevalence and spatiotemporal properties of PIP_2_ dynamics in central neurons under physiological conditions. PIP_2_ levels and their dynamic regulation may be largely different *in vivo*, as embedding in the native environment and full differentiation of neurons may impact on relevant factors such as expression and spatial subcellular organization of receptors and downstream components of the signaling cascade and the enzymes that resynthesize PIP_2_. Therefore, information on PIP_2_ concentration behavior in intact tissue preparations such as brain slices is required. Previous studies with organotypic slices were consistent with PIP_2_ depletion *in-situ* triggered by synaptic release of glutamate onto cerebellar Purkinje neurons^75^ or by muscarinic agonist application in cortical pyramidal cells^76^. However, both studies were not fully conclusive with respect to PIP_2_ signaling because PLCδ1-PH was used as a sensor domain, which has a similar affinity for PIP_2_ and IP_3_ and may report the production of IP_3_ rather than depletion of PIP_2_^44,77^. In fact, Okubo *et al*.^75^ interpreted probe translocation in terms of IP_3_ production rather than depletion of PIP_2_.

Here, by using tubby_CT_, as an alternative PIP_2_ sensor insensitive to IP_3_^33,34,40^, our present data now show unequivocally that muscarinic and metabotropic glutamate receptors indeed trigger PIP_2_ dynamics in a prototypic central neuron *in situ*. Of note, in cultured cell lines tubby_CT_ previously failed to respond or only weakly translocated upon PLC-mediated PIP_2_ depletion^34,40^, which initially was attributed to a higher affinity for PIP_2_ compared to PLCδ1-PH. However, quantitative titration of PIP_2_ in living cells showed that its affinity to PIP_2_ is actually lower, which should make it a useful PIP_2_ sensor^41^. In fact, our current results demonstrate that tubby_CT_ readily translocates in a neuronal cellular environment. The difference in behavior between experimental conditions is not yet understood, but may suggest cell type-specific segregation of PIP_2_ into distinct pools selectively accessible to the different PIP_2_-binding domains^78^. In any case, our findings show that in the native neuronal system tubby_CT_ is a much better reporter of PIP_2_ dynamics than might have been anticipated^20^. Thus, using tubby_CT_-GFP allowed us to systematically assay PIP_2_ dynamics without confounding effects of IP_3_ dynamics.

### Spatiotemporal properties of PIP_2_ dynamics

We found that in the larger compartments accessible to measuring sensor translocation, receptor-induced PIP_2_ depletion appeared largely homogenous without evidence for substantial subcellular differences. This suggested that induced PLC activity is similar in somatic and dendritic compartments. This finding was not entirely expected, because while M1 receptors show high densities throughout soma and dendrites^73^, the most prominent mGluRI receptor of CA1 neurons, mGluR_5_, has a relatively low density in soma compared to dendrites^47^. Since glutamatergic PIP_2_ depletion in dendrites was not stronger than in the somatic compartment, the PIP_2_ depletion pattern does not appear to correlate closely with receptor distribution. It is worth noting that the moderate degree of PIP_2_ sensor translocation in dendrites did not result from the distinct dendritic geometry, because the smaller volume-to-membrane area ratio in the dendrites should rather result in a stronger relative increase of sensor fluorescence when sensors dissociate from the membrane. Also, muscarinic stimulation elicited larger dendritic responses than glutamatergic stimulation, showing that PIP_2_ sensor response was not saturated by mGluR stimulation. Of note, a similar observation was made by Nakamura *et al*.^79^ for Ca^2+^ dynamics in CA1 pyramidal cells: activation of mGluRI, mAChR and 5-HT_2_R elicited comparable Ca^2+^ waves despite different receptor distribution. Thus, these neurons may possess mechanisms to globalize Gq signaling including PIP_2_ depletion.

To our knowledge our results for the first time demonstrate oscillations of the PIP_2_ concentration in a neuron. While IP_3_, DAG and Ca^2+^ are known to undergo oscillatory concentration dynamics in neurons^80^, previous observations on PIP_2_ dynamics in primary dissociated neurons^16,18,33^ seemed to indicate that PIP_2_ concentrations essentially remained depleted during prolonged receptor activity. Oscillatory translocation of the PLCδ1-PH sensor domain observed occasionally has been understood as dynamics of the IP_3_ signal picked up by the PH domain^23,44,81^. More recently, careful observations also including the specific tubby_CT_ sensor showed bona-fide oscillations of PIP_2_ in mast cells^82,83^. Our observations suggest that such dynamics may be a more general phenomenon with implications for neuronal biology.

The mechanisms underlying PIP_2_ oscillations may include both positive and negative feedback regulation of PIP_2_ cleavage by PLC. Such mechanisms have previously been shown to be involved in Ca^2+^ and IP_3_ oscillations and include Ca^2+^-dependent activation of PLC^80,81^ as a positive feedback. In mast cells, PIP_2_ oscillations are probably driven by Ca^2+^ oscillations^82^. Inhibition of Gq signaling by, e.g., PKC, receptor kinases, or RGS molecules^81,84^ may contribute to a negative feedback loop controlling PIP_2_ degradation. Moreover, our observations reveal an impressive capability of PIP_2_ replenishment, as indicated by the rapid and complete recovery of PIP_2_ levels in presence of agonists. PIP_2_ resynthesis may be increased during GqPCR activation^85^, providing negative feedback to PIP_2_ depletion and possibly contributing to observed oscillations. Specifically, PIP_2_ replenishment may involve Ca^2+^ and phosphatidic acid-dependent phospholipid exchange at plasma membrane-endoplasmic reticulum (PM-ER) junctions^86^.

Whatever the mechanism underlying the oscillations is, our findings indicate that PIP_2_ dynamics may provide neurons with another dimension of effector modulation beyond a simple on/off switch for downstream effectors. Although the consequences of PIP_2_ oscillations for electrical neuronal activity remain to be explored, we note that indeed, neurons showed fluctuations of membrane potential and firing frequency during agonist application. It is worth mentioning that mAChR and mGluRI agonists can induce and shift gamma and theta oscillations^87^. In the light of the present data, it is tempting to speculate that PIP_2_ oscillations might participate in such frequency modulation.

### Neuronal ion channels as effectors of PIP_2_ dynamics

Given the known high sensitivity of some ion channels to even a moderate drop in the PIP_2_ concentration^5,78^, a main potential target of PIP_2_ depletion are ion channels and thus electric excitability. Based on studies on isolated neurons, inhibition of Kv7 channels in sympathetic neurons as the direct consequence of PIP_2_ depletion is well established^13,17,29^. Our data permit the correlation of PIP_2_ dynamics and electrophysiology *in situ*. Activation of mAChR and mGluRI, but not other PLC-coupled receptors known to be present and functional in CA1 neurons induced robust PIP_2_ depletion. The same pattern of receptor specificity was observed for modulation membrane potential, firing frequency and afterhyperpolarization, providing at least circumstantial evidence for the causation of channel regulation by PIP_2_. Simultaneous recordings of electrical activity and PIP_2_ dynamics from the same neuron should be performed in the future to provide more direct evidence.

In conclusion, our data support and generalize the as yet largely hypothetical mechanism of PIP_2_ dynamics as a major cellular signal in the control of neuronal activity through regulation of PIP_2_-sensitive ion channels such as Kv7. Future studies need to address this issue rigorously by manipulating PIP_2_ levels *in-situ*^20^. Along those lines a recent study aimed at PIP_2_ depletion in hippocampal slice cultures by chemically induced recruitment of a PIP_2_ phosphatase^88^. While this approach did not reveal any effects on electrical properties of the neurons, the results appear inconclusive since changes in PIP_2_ concentration were not verified.

One of the most intriguing unknowns are the spatiotemporal properties of PIP_2_ dynamics during entirely physiological neuronal activity, i.e, during synaptic activity of the modulatory (e.g. cholinergic) and principal (i.e. glutamatergic) inputs into the hippocampal neurons and of the PIP_2_ dynamics associated with intrinsic neural (network) activity. Another question is the PIP_2_ signaling in the distal smaller dendritic compartments not amenable to analysis by the translocation probes used in this study. In particular, in the immediate postsynaptic compartment, i.e. spines, PIP_2_ may have a role in controlling synaptic plasticity^89–91^.

## Materials and Methods

### Virus production and constructs

Lentiviral plasmids pCMVΔR8.9, pVSVG and FUGW were kindly provided by Pavel Osten (MPI for medical research Heidelberg, Germany). The PLCδ1-PH and tubby_CT_ constructs were provided by Tamás Balla (NIH, Bethesda, USA) and Lawrence Shapiro (Columbia University, USA), respectively. Lentiviral particles were derived by triple transfection of HEK293FT cells with Lipofectamin 2000 (Invitrogen, Darmstadt, Germany). Virus purification from supernatant was achieved by 15 minute centrifugation at 3000 rpm, filtration through a Millex ® HV 0.45 µm filter (Millipore, Darmstadt, Germany) and two successive ultracentrifugation steps (25000 rpm, 1 h 30 min, 4 °C). Pellets were resuspended in TBS-5 buffer (50 mM Tris-HCl, 130 mM NaCl, 10 mM KCl, 5 mM KCl_2_) and subjected to a final 30 s centrifugation at 5000 rpm. Aliquots were stored at −80 °C and thawed up to two times.

### Animals, stereotactic injection and slice preparation

Wistar rats were obtained from the animal facility of the Philipps University of Marburg (Marburg, Germany) or Charles River (Cologne, Germany) and kept and handled according to German law and institutional guidelines at the Philipps University. All procedures were approved by the Regierungspräsidium Giessen, Germany. Animals were housed with access to *ad libitum* water and food on a 12-h light/dark cycle. At weaning (postnatal day 21) male and female rats were anesthetized by intraperitoneal injection of a mixture of ketamine (Bela-Pharm, Vechta, Germany) and xylazine (Rompun®, Bayer AG, Leverkusen, Germany) at a dose of 100 and 10 mg per kilogram body weight. Additionally, the mixture included 0.05 mg/kg Atropine (B. Braun, Melsungen, Germany) and 0.1 ml/10 g body weight of a 0.9% NaCl solution for injection (Diaco, Triest, Italy). Under stereotactic control, lentivirus was injected bilaterally using Paxinos and Watson^92^ as a reference. Coordinates were optimized for targeting in juvenile rats by setting the adult references to x = +/−6.125, y = −6.15 and z = −6.2 mm and multiplying by the ratio of the juvenile to atlas (8.7 mm) distance of bregma to lambda. Up to 2.5 μl virus per hemisphere were injected in 500 nl portions going from ventral to dorsal in 0.3–0.35 mm steps during 5–10 minutes. For imaging and electrophysiological experiments, rats were anesthetized with Isoflurane (Baxter, Unterschleißheim, Germany) or Sevoflurane (Sevorane®, Abbott, Wiesbaden, Germany) and sacrificed by decapitation at the ages indicated in results. The head was placed in ice cold sucrose-ACSF (sucrose-artificial cerebrospinal fluid, in mM: 87 NaCl, 25 NaHCO_3_, 25 D-glucose, 75 sucrose, 2.5 KCl, 0.5 CaCl_2_ and 7 MgCl_2_, oxygenated with 95% O_2_/5% CO_2_) and the hippocampi rapidly removed. 300 μm transversal slices were cut with a vibratome (VT1200, Leica Biosystems, Wetzlar, Germany) and placed into a chamber with 4 °C sucrose-ACSF. After a 35 min recovery period at 35 °C slices were kept at room temperature. For recordings slices were transferred to a submerged chamber and perfused with ACSF (in mM: 125 NaCl, 25 NaHCO_3_, 25 D-glucose, 2.5 KCl, 2 CaCl_2_ and 1 MgCl_2_, oxygenated with 95% O_2_/5% CO_2_) for at least 20 minutes.

### Imaging, electrophysiological recording and data analysis

Confocal imaging was performed with a Zeiss LSM710 (Zeiss, Oberkochen, Germany). The sampling rate for time series experiments was 1.75 s with a pixel size of 0.13 µm. In some cases (especially dendrite measurements) the sampling rate was increased to 1 s. In all cases where the sampling rate slightly deviated the data were resampled to allow averaging across experiments. Overlay with the original was performed to ensure preservation of time scale. Average cytoplasmic fluorescence intensities were determined from regions of interest (ROI) excluding both the plasma membrane (defined as the local intensity max at the cell’s border in the resting cell) and the nucleus. Distance of ROIs to the plasma membrane was >0.5 µm even when slight shifts of the cell’s position occurred during the experiment. ROIs were defined post-hoc using the microscope software ZEN (Versions 2008 and 2009) and obtained average intensities were exported to Igor Pro (Version 6.03 A, Wave Metrics, Portland, OR USA). Traces were background subtracted and normalized to the last time point before beginning of a response (F/F_0_ normalized to t_0_). Measurements without evident response were corrected for photobleaching according to a biexponential fit to the decaying fluorescence signal and normalized to signal at the onset of agonist application. We found that probe translocation generally prevented the reliable determination of the time course of photobleaching. Therefore most data were not corrected for bleaching which results in apparently lower signals following transient depletion of PIP_2_, with bleaching generally being more pronounced for tubby_CT_-GFP than for PLCδ1-PH-GFP. Confocal images were further analyzed with ImageJ (National Institutes of Health, USA) to isolate individual images of a time series, create kymographs and set scale bars. Electrophysiological data were recorded with a HEKA EPC10USB amplifier and Patch Master software (Version 2.43 HEKA, Lambrecht, Germany) in current clamp mode. Data were low pass filtered with a 2.9 kHz Bessel filter and digitized at 20 kHz. Borosilicate recording pipettes had a resistance of 3–4 MΩ and were filled with intracellular solution containing (in mM): K-gluconate 135, KCl 20, MgCl_2_ 2, Na_2_-ATP 2, Na_2_-GTP 0.3, HEPES 10 and EGTA 0.1 (adjusted to pH 7.2 with KOH). Series resistance was monitored in voltage clamp mode before and after each current clamp recording, but not corrected for. Measurements with a change in series resistance >40% during the course of the experiment were discarded. Input resistance was assessed by injection of small positive and negative currents steps, followed by a depolarizing current step above action potential threshold to quantify spiking behavior and afterpolarisation (see Fig. 5a,b). Sweep length was 7 seconds. In applications of the P2Y_1_ agonist ADPβS a shorter protocol without the positive 20 pA step was used. Medium afterhyperpolarisation (AHP_m_) was obtained as the difference of resting V_m_ and mean V_m_ at 70 to 120 ms after the depolarizing current step. Changes in AHP value resulting from application of receptor agonists are given as Δafterpolarization such that positive values indicate reduction of AHP or eventually the emergence of an afterdepolarisation. Amplitudes were calculated from averaging at least 10 baseline data points and a minimum of 3 peak points, with avoidance of plateau potentials.

### Statistical analysis

Statistical significance was tested in Igor Pro. Randomness, equal variances and normal distribution of the data was tested with Igor’s Runs, Kolmogorow-Smirnow and Jarque-Bera test. In cases where validity of a parametric test was compromised, a Wilcoxon-Mann-Whitney test was performed. Where applicable, groups of two were compared with paired and unpaired Student’s t. Two tailed one-way ANOVA was followed by a Dunett test for comparing multiple groups to a single control or a Tukey test to compare all groups to each other. Unless noted otherwise all values are given ± standard error of the mean.

### Chemicals and perfusion system

Oxotremorine-M, DHPG, Bradykinin, SKF83959, DOI, Serotonin and Dopamine were purchased from Tocris and Methoxamine and 2-Pyridylehylamin from Sigma. All other chemicals were from Sigma/Fluka or Merck (Germany). For application of test substances a capillary of 200 to 250 µM inner diameter (TSP200350, BGB Analytik AG, Boeckten, Germany or MicroFil MF28G-5, World Precision Instruments, Berlin, Germany) was placed directly next to the hippocampal recording region. Solution exchange at the tip occurred within 1–2 s. Unless noted otherwise recordings represent first applications of each test substance. To block fast glutamatergic and GABAA/B signaling in electrophysiological recordings, receptor antagonists (4 μM NBQX, 50 μM D-AP5, 50 μM Picrotoxin and 1 μM CGP 55845, all from Tocris) were added both to the bath and local perfusion.

## Data Availability

Most data generated or analysed during this study are included in this published article. Additional datasets generated and analysed during the current study are available from the corresponding authors on reasonable request.
